# Gut microbiome alters functions of mutant p53 to promote tumorigenesis

**DOI:** 10.1038/s41392-020-00336-y

**Published:** 2020-10-09

**Authors:** Harry Cheuk Hay Lau, Jun Yu

**Affiliations:** Institute of Digestive Disease, Department of Medicine and Therapeutics, State Key Laboratory of Digestive Disease, Li Ka Shing Institute of Health Sciences, CUHK Shenzhen Research Institute, The Chinese University of Hong Kong, Sha Tin, N.T. Hong Kong

**Keywords:** Oncogenes, Gastrointestinal cancer

In a recent study in *Nature*, Kadosh et al.^1^ established a landmark relationship between the gut microbiome and host epigenetics in intestinal oncogenesis. They demonstrated the substantial plasticity of mutant p53 in WNT-driven tumorigenesis, and the crucial involvement of gut microbiome in modulating this plasticity.

**Fig. 1:**
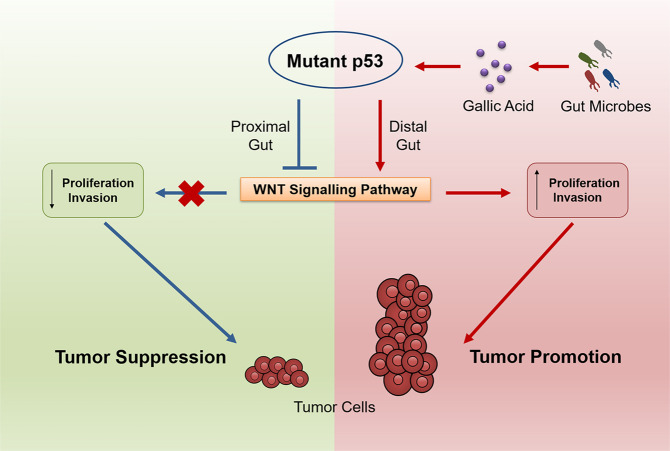
Mutant p53 inhibits tumorigenesis by avoiding the initiation of oncogenic WNT signaling pathway. However, when mutant p53 is exposed to gallic acid, which is a polyphenol metabolite produced by gut commensal microbes, it loses its tumor-suppressive property and switches to perform oncogenic functions by over-activating WNT signaling pathway to enhance proliferation and invasion of tumor cells

Human gastrointestinal tract harbors a diverse microbial community that stably and continuously interacts with host cells to perform physiological functions. Yet, dysbiosis—a perturbation in the compositional and/or functional profiles of microbiome, is closely linked with the progression of colorectal cancer (CRC). In particular, enrichments of CRC-associated microbes (e.g., *Fusobacterium nucleatum*, which activates nuclear factor-κB pathway to generate a pro-inflammatory tumor microenvironment) together with their metabolic derivatives in gut are known to contribute intestinal tumorigenesis by inducing chronic inflammation and DNA damage.^2^ Meanwhile, to date there is inadequate study to investigate the association between microbiome and host epigenetics in CRC. In a recent study of Kadosh et al.,^1^ they reported that the gut microbiome could actually affect the functions of mutant p53 to promote intestinal tumor formation.

It is widely accepted that mutations in p53 are predisposed to cancer development, progression, and metastasis. However, growing evidence has illustrated that distinct mutations in p53 can have differing properties, and some of them even exhibit tumor-suppressive features.^3^ As a team that previously discovered the inhibitory effects of wild-type p53 on the oncogenic WNT signaling pathway, Kadosh and colleagues proceeded to ascertain the role of mutant p53 in WNT-driven tumorigenesis. They introduced mutations R172H and R270H (R175H and R273H in humans, respectively, which are two hotpot mutations in p53) into the endogenous *p53* locus in mice. Mice were then bred to homozygosity with two transgenic mouse models of WNT-driven intestinal cancer—mice with gut-inducible deletion of *CKIa* (encoding a component protein of the β-catenin–destruction complex) and the canonical *Apc*^*Min/+*^ mice.

Kadosh and colleagues first examined the cancer phenotypes in the proximal (duodenum and jejunum) and distal (ileum and colon) gastrointestinal tract of *CKIa*-deleted mice carrying wild-type *p53* (*CKIa*^Δ*gut*^), mutant *p53* (*CKIa*^Δ*gut*^*p53*^*R172H*^), or no *p53* (*CKIa*^Δ*gut*^*p53*^Δ*gut*^). In humans, both mutations in p53 and WNT hyperactivation are common in CRC.^4^ Indeed, the colonic epithelia in *CKIa*^Δ*gut*^*p53*^*R172H*^ mice were highly dysplastic with enhanced WNT activity. Surprisingly, the jejunum in *p53*^*R172H*^ mutant mice showed normal level of cell proliferation and no evidence of invasion. Such opposing phenotypes at different gut segments were absence in mice from other groups. The authors then bred *p53*^*R172H*^ mutant mice with *Apc*^*Min/+*^ mice (a model known for frequent appearance of intestinal tumors but with rare colonic tumorigenesis) for validation. Again, the tumor incidence in colon was markedly increased, while the tumor burden in jejunum was attenuated. These data obviously implicated the paradoxical effect of mutant p53 on WNT-driven cancer—suppressing tumorigenesis in the proximal gut, but enhancing tumorigenesis in the distal gut.

Next, Kadosh and colleagues deciphered the underlying mechanisms by examining the expressions of WNT target genes, including Cd44, cyclin D1, and Myc. In line with the phenotypes, expressions of these genes were significantly lowered in the jejunum of *p53*^*R172H*^ mutant mice. By contrast, their expressions in the distal gut were high with levels similar to those observed in p53-knockout mice. To evaluate why mutant p53 could not suppress tumorigenesis in the distal gut, Kadosh and colleagues cultured organoids derived from ileum of *CKIa*-deleted mice. Under this in vitro setting, ileal organoids from *CKIa*^Δ*gut*^*p53*^*R172H*^ mice exhibited moderate WNT activity and cell proliferation. While ileal organoids from mice with double-knockout of *CKIa* and *p53* illustrated WNT hyperactivation, hyperproliferation, and poor differentiation. The authors further overexpressed human mutant p53 (R175H and R273H) in these double-knockout organoids. Interestingly, differentiation in these human mutant-p53-expressing ileal organoids was now restored with re-organization of proliferated cells.

Since the ileum-derived organoids from *p53*^*R172H*^ mutant mice were able to exhibit inhibitory effects on WNT signaling pathway, it is rational to say that this mutant p53 can be protective against tumorigenesis. Yet, the heterogeneity on in vivo phenotypes suggested that some additional factors inside the gut yielded the ability to override the tumor-suppressing feature of mutant p53. Apart from the general structure and function, the microbiome is known to be distinct between proximal and distal gut, as accumulated studies have reported that each gut segment has its own microbial profile. Kadosh and colleagues, therefore, hypothesized that the microbiome might be responsible to counteract mutant p53-mediated suppression on WNT activity in the distal intestine, and they tested this assumption by treating *CKIa*^Δ*gut*^*p53*^*R172H*^ mice with antibiotics to eliminate their commensal microbes. Indeed, these antibiotic-treated mice had reduced WNT activation and decreased cell proliferation specifically in their ileum and colon.

Metabolism is a key area where the host and microbiota interact, and metabolites are generated when gut microbes metabolize dietary nutrients. Notably, several major subgroups of metabolites have been correlated with colorectal tumorigenesis, including short-chain fatty acids, lipid derivatives and polyphenols.^5^ Kadosh and colleagues, therefore, speculated that metabolites in the distal gut might have the potential to counteract the WNT-suppressive effect of mutant p53. They treated jejunal organoids derived from *CKIa*^Δ*gut*^*p53*^*R172H*^ mice with a panel of metabolites, and identified that gallic acid (a polyphenol produced by *Lactobacillus plantarum* and *Bacillus subtilis*, which secrete shikimate dehydrogenase to degrade 3-hydroxyshikimate into gallic acid) significantly increased proliferation and WNT activity in organoids. Upon removal of gallic acid, these jejunal organoids lost their hyper-WNT-proliferative properties and reverted back to the normal organoid appearance, hence indicating that continuous presence of gallic acid was necessary to avoid the tumor-suppressive feature of mutant p53. The authors then supplemented gallic acid to *p53*^*R172H*^ mutant mice and high-grade dysplasia in the proximal gut was displayed, thus validating the observations on in vitro cultures. In addition, two essential epigenetic features for enabling WNT transcription (TCF4–β-catenin interaction and H3K4me3 histone modification) were greatly promoted in the jejunal enterocytes of gallic acid-treated *p53*^*R172H*^ mutant mice, thereby leading to enhanced expressions of WNT target genes.

Collectively, Kadosh and colleagues demonstrated the functional plasticity of mutant p53 in WNT-driven tumorigenesis, and the critical involvement of gut microbiome in modulating this plasticity (Fig. 1). Of note, as the effect of gallic acid on mutant p53 could be reversed easily, it is, therefore, plausible to treat cancer by targeting microbiome (e.g., dietary management or use of gallic acid antagonist). In summary, this paper establishes a landmark relationship between microbiome and host epigenetics in intestinal tumorigenesis, and extensive work is urgently needed to elaborate this relatively less-studied area.

## References

[1] Kadosh E (2020). The gut microbiome switches mutant p53 from tumour-suppressive to oncogenic. Nature.

[2] Wong SH, Yu J (2019). Gut microbiota in colorectal cancer: mechanisms of action and clinical applications. Nat. Rev. Gastroenterol. Hepatol..

[3] Sabapathy K, Lane DP (2018). Therapeutic targeting of p53: all mutants are equal, but some mutants are more equal than others. Nat. Rev. Clin. Oncol..

[4] Guinney J (2015). The consensus molecular subtypes of colorectal cancer. Nat. Med..

[5] O’Keefe SJD (2016). Diet, microorganisms and their metabolites, and colon cancer. Nat. Rev. Gastroenterol. Hepatol..

